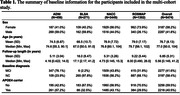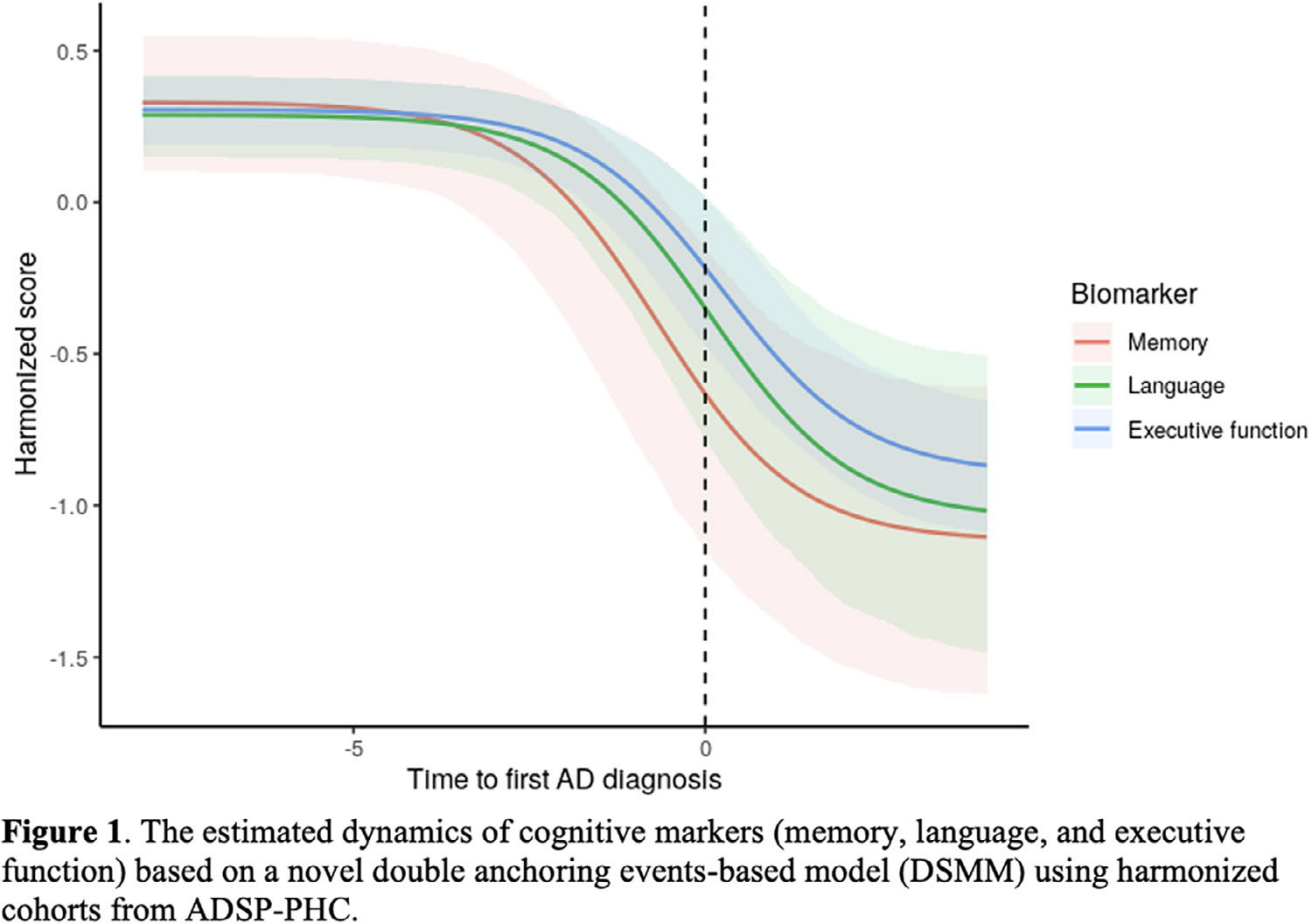# Comparing the dynamics of multiple cognitive markers for Alzheimer’s disease progression using a novel double anchoring events‐based sigmoidal mixed model

**DOI:** 10.1002/alz.085501

**Published:** 2025-01-09

**Authors:** Kaidi Kang, Panpan Zhang, Logan C. Dumitrescu, Shubhabrata Mukherjee, Michael L. Lee, Seo‐Eun Choi, Phoebe Scollard, Laura E. Gibbons, Emily H. Trittschuh, Jesse Mez, Andrew J. Saykin, Rachel F. Buckley, Xiaoting Gao, Jianing Di, Paul K. Crane, Timothy J. Hohman, Dandan Liu

**Affiliations:** ^1^ Department of Biostatistics, Vanderbilt University Medical Center, Nashville, TN USA; ^2^ Vanderbilt Memory & Alzheimer’s Center, Vanderbilt University Medical Center, Nashville, TN USA; ^3^ Department of Neurology, Vanderbilt University Medical Center, Nashville, TN USA; ^4^ Vanderbilt Genetics Institute, Institute for Medicine and Public Health Vanderbilt University Medical Center, Nashville, TN USA; ^5^ University of Washington, Seattle, WA USA; ^6^ Department of Medicine, University of Washington, Seattle, WA USA; ^7^ University of Washington, School of Medicine, Seattle, WA USA; ^8^ Department of Psychiatry and Behavioral Sciences, University of Washington School of Medicine, Seattle, WA USA; ^9^ Boston University Alzheimer’s Disease Research Center, Boston University Chobanian & Avedisian School of Medicine, Boston, MA USA; ^10^ Indiana Alzheimer’s Disease Research Center, Indianapolis, IN USA; ^11^ Brigham and Women’s Hospital and Department of Neurology, Massachusetts General Hospital, Harvard Medical School, Boston, MA USA; ^12^ Janssen China Research and Development, Shanghai China; ^13^ Vanderbilt Genetics Institute, Vanderbilt University Medical Center, Nashville, TN USA

## Abstract

**Background:**

Understanding the dynamics of markers throughout Alzheimer’s disease (AD) progression in a representative population is critical for early detection of AD. Most existing studies used a single cohort to model the dynamics of AD‐related markers, which may lead to biased and unreproducible results. The Alzheimer’s Disease Sequencing Project Phenotype Harmonization Consortium (ADSP‐PHC) harmonized rich endophenotype data across multiple cohort studies, providing valuable resources for ADRD research. Using harmonized cognitive markers from ADSP‐PHC, we modeled and compared dynamics of memory, language, and executive function over AD progression through a novel double anchoring events‐based sigmoidal mixed model (DSMM).

**Method:**

The study included diagnostically irreversible participants with clinical diagnoses of mild cognitive impairment (MCI) and/or AD (n = 5,475; Table 1). A novel DSMM used time to the first diagnosis of AD as time scale, and extended the standard sigmoidal mixed model to include the participants with unobserved AD diagnosis by jointly modeling the duration between the first diagnoses of MCI and AD. The model provided inference about initial score, maximum decline (of score) and half‐life of decline (time from when score dropped by half) for the population. A log‐linear model characterizing the duration between clinical diagnoses of MCI and AD was adjusted for age (at MCI diagnoses), sex, apolipoprotein E‐ε4 status, and their interactions.

**Result:**

The three cognitive markers have similar initial scores (memory: 0.33, 95%CI: 0.08‐0.54; language: 0.29, 95%CI: 0.14‐0.41; executive function: 0.31; 95%CI: 0.18‐0.40). Memory has the largest decline (β = 1.45, 95%CI: 1.01‐1.90), followed by language (β = 1.33, 95%CI: 0.81‐1.82) and executive function (β = 1.20, 95%CI: 1.01‐1.39). The half‐life of decline is the shortest for memory (β = ‐0.69, 95%CI: ‐1.72‐0.34), followed by language (β = 0.08, 95%CI: ‐0.88‐1.01) and executive function (β = 0.26, 95%CI: ‐0.41‐0.94; Figure 1).

**Conclusion:**

A novel DSMM was used to characterize the dynamics of multiple harmonized cognitive markers related to AD progression using multiple harmonized cohorts from ADSP‐PHC, empirically determining the temporal order of decline of harmonized cognitive markers: memory, then language, and lastly executive function. The study improved the representativeness of the research sample by using multiple cohorts and harmonized cognitive markers, and DSMM helped reduce selection bias by including participants without observed clinical diagnosis of AD.